# Evidence of Association between CTLA-4 Gene Polymorphisms and Colorectal Cancers in Saudi Patients

**DOI:** 10.3390/genes14040874

**Published:** 2023-04-06

**Authors:** Nouf Al-Harbi, Maha-Hamadien Abdulla, Mansoor-Ali Vaali-Mohammed, Thamer Bin Traiki, Mohammed Alswayyed, Omar Al-Obeed, Islem Abid, Suliman Al-Omar, Lamjed Mansour

**Affiliations:** 1 Department of Zoology, College of Science, King Saud University, Riyadh 11472, Saudi Arabia; noufnalharbi@gmail.com (N.A.-H.);; 2 Department of Surgery, College of Medicine, King Saud University, Riyadh 11472, Saudi Arabia; mansooralicytogene@gmail.com (M.-A.V.-M.);; 3 Department of Pathology and Laboratory Medicine, College of Medicine, King Saud University, Riyadh 11495, Saudi Arabia; 4 Department of Botany and Microbiology, Science College, King Saud University, Riyadh 11495, Saudi Arabia

**Keywords:** colorectal cancer, CTLA-4, SNP polymorphism, rs231775, rs3087243, check point molecules, haplotypes, gene expression

## Abstract

Cytotoxic T lymphocyte antigen-4 (CTLA-4) has been identified as an immunosuppressive molecule involved in the negative regulation of T cells. It is highly expressed in several types of autoimmune diseases and cancers including colorectal cancer (CRC). (1) **Objective:** To explore the association between CTLA-4 single nucleotide polymorphisms (SNP) and risk to (CRC) in the Saudi population. (2) **Methods:** In this case-control study, 100 patients with CRC and 100 matched healthy controls were genotyped for three CTLA-4 SNPs: rs11571317 (−658C > T), rs231775 (+49A > G) and rs3087243 (CT60 G > A), using TaqMan assay method. Associations were evaluated using odds ratios (ORs) and 95% confidence intervals (95% CIs) for five inheritance models (co-dominant, dominant, recessive, over-dominant and log-additive). Furthermore, CTLA-4 expression levels were evaluated using quantitative real-time PCR (Q-RT-PCR) in colon cancer and adjacent colon tissues. (3) **Results:** Our result showed a significant association of the G allele (OR = 2.337, *p* < 0.0001) and GG genotype of the missense SNP +49A > G with increased risk of developing CRC in codominant (OR = 8.93, *p* < 0.0001) and recessive (OR = 16.32, *p* < 0.0001) models. Inversely, the AG genotype was significantly associated with decreased risk to CRC in the codominant model (OR = 0.23, *p* < 0.0001). In addition, the CT60 G > A polymorphism exhibited a strong association with a high risk of developing CRC for the AA genotype in codominant (OR = 3.323, *p* = 0.0053) and in allele models (OR = 1.816, *p* = 0.005). No significant association was found between −658C > T and CRC. The haplotype analysis showed that the G-A-G haplotype of the rs11571317, rs231775 and rs3087243 was associated with high risk for CRC (OR = 57.66; *p* < 0.001). The CTLA-4 mRNA gene expression was found significantly higher in tumors compared to normal adjacent colon samples (*p* < 0.001). (4) **Conclusions**: Our findings support an association between the CTLA-4 rs231775 (+49A > G) and rs3087243 (CT60 G > A) polymorphisms and CRC risk in the Saudi population. Further validation in a larger cohort size is needed prior to utilizing these SNPs as a potential screening marker in the Saudi population.

## 1. Introduction

Cancer is the leading cause of death worldwide, with approximately 10 million deaths in 2020 [1,2]. Colorectal cancer (CRC) represents one of the major causes of cancer-related mortalities in Western countries and, along with lung, prostate and breast cancer, is among the most common malignancies [3,4]. In 2021, the number of new cases in Saudi Arabia was 27,885 including 4007 colorectal cancer cases, accounting for 14.4% of the total cases [5]. Cancer is a complex disease involving interactions between environmental factors and genetic variations [6,7,8]. Chemotherapy and targeted therapy approaches have extended the survival rate for patients with unresectable CRCs; however, side effects and drug resistance are considered major challenges, which are mainly due to tumor heterogeneity [6,9]. Over the past years, several studies have reported the complex relationships between cancer and the immune system which set the foundation of immunotherapy approach [10,11]. The immune system has a highly regulated surveillance mechanism that enables the control of tumor growth. The maintenance of regulated immune responses by both co-stimulatory and co-inhibitory signaling is crucial for cancer control [12]. One of the most studied checkpoint molecules is cytotoxic T lymphocyte antigen-4 (CTLA-4) [13]. CTLA-4 or CD152 is a member of the immunoglobulin superfamily that is expressed on the surface of T lymphocytes and functions as a T cell activation negative regulator. CTLA-4 has a similar binding affinity for both costimulatory receptors B7-1 (CD80) and B7-2 (CD86) on antigen presenting cells (APC) and through this interaction it produces an inhibitory signal that suppress T cell activation [14]. The study of immune-related genetic markers, especially genes involved in regulating immune responses, is important in understanding the role of the immune system in tumor progression and resorption. Single nucleotide polymorphisms (SNPs) in immune-related genes may be involved in altered immune responses to cancer [15]. In recent years, polymorphisms in the immune checkpoint genes have been heavily investigated to reveal their relations to several malignancies including CRC [16,17,18,19]. In fact, there is increasing focus on SNPs and their involvement in individuals’ susceptibility to several diseases, including solid tumors. Large-scale genome-wide association studies have identified different loci associated with CRC [20,21,22,23]. The CTLA-4 gene contains multiple SNPs that can affect gene expression, and lead to amino acid changes and changes in mRNA splicing, which could influence T cell activity leading to the attenuation of immune response [24]. It has been reported that SNPs in the CTLA-4 gene were linked to various types of malignancies including breast [25,26,27,28], cervical [29,30,31,32,33,34] and lung cancers [19,27,35,36] and oral squamous cell carcinoma [37,38]. Additionally, several studies have shown that CTLA-4 variants affect CTLA-4 expression on the cell surface, which was associated with autoimmune diseases, such as Grave’s disease, Hashimoto’s thyroiditis and atopic dermatitis [39,40,41,42]. Among the CTLA-4 variants, three were reported for their functional effect on the gene expression or protein efficiency and were found associated with many diseases. These SNPs include: CT60 in the 3′-UTR (rs3087243), the missense variant 49AG (rs231775) resulting in a threonine to alanine amino acid change at codon 17 (T17A) of the leader peptide, and −658C > T (rs11571317) located in the promoter region. Therefore, this study investigated the relationships between these three functional polymorphisms and their association with CRC in the Saudi Arabian population and the assessment of the expression profile of CTLA-4 in cancer tissue.

## 2. Materials and Methods

### 2.1. Study Population

Two hundred blood samples were obtained from King Khalid University Hospital. A total of 100 patients with sporadic colorectal cancer and 100 healthy controls with no history of cancer were included. Colorectal cancer patients comprised patients of diverse ages (mean age  =  57 years) and three stages of the disease. Blood samples were collected from patients preoperatively. Patients did not undergo irradiation treatment or chemotherapy. Determination of CRC was made utilizing standard diagnostic methods and confirmed by histopathological methods. All subjects including patients and controls were of Saudi Arabian ethnicity. Healthy controls were gender- and age-matched and enrolled after diagnostic exclusion of cancer. The study fulfilled its requirements and has been approved by the Ethics Committee of the King Saud University. Patient consent was acquired for this study, IRB00008189. This study was conducted in accordance with the Declaration of Helsinki.

### 2.2. DNA Extraction

Approximately 3 mL blood samples were collected in vacutainers containing ethylenediaminetetraacetic acid (EDTA) from all subjects registered in the study. Genomic DNA was extracted from peripheral blood using the QIAamp DNA Blood Mini Kit (Qiagen, Valencia, CA, USA) and stored at −80 °C until used. Following extraction and purification, the DNA was quantitated spectrophotometrically on NanoDrop 8000 (Thermo Scientific, Wilmington, DE, USA), and examined using standard A260/A280 and A260/A230 ratios to test its purity.

### 2.3. Preparation of Total RNA and qRT-PCR

RNA was extracted from 31 tumor and from 31 cancer-free margins of the adjacent tissue from cancer patients using a PARIS™ kit (Ambion, Foster City, CA, USA). A High-Capacity cDNA kit was used for reverse transcription (cat. no. 4368814; Applied Biosystems, Foster City, CA, USA). The quality of RNA was evaluated by assessing the A260/280 ratio (1.8–2.0). To remove the contamination of genomic DNA, RNA samples were treated with RNAse-free DNA enzyme (Ambion, Foster City, CA, USA). The final RNA (2 μg) concentrations in each analyzed sample were subjected to the RT reaction. The reverse transcription reaction was performed using a commercially available set of high capacity cDNA Archive Kits (Applied Biosystems, Waltham, MA, USA). cDNA was prepared from 2 μg of total RNA, with random hexamer primers. According to the manufacturer’s instructions the mix was run on a PCR thermocycler gene as follows: 10 min at 25 °C, 2 h at 37 °C, 5 min at 85 °C and kept at 4 °C thereafter on a PCR thermocycler (Applied Biosystems, Waltham, MA, USA).

### 2.4. Quantitative Real-Time Reverse Transcription-Polymerase Chain Reaction (qRT-PCR)

**Figure 1:**
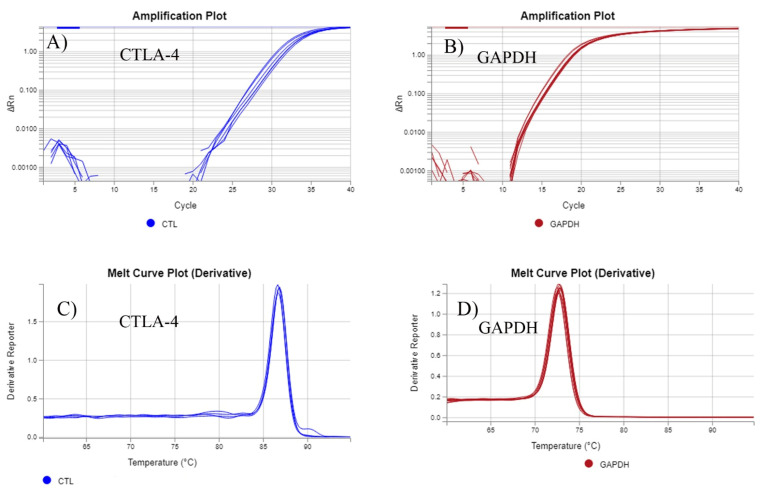
Amplification curves (**A**,**B**) and melting curves (**C**,**D**) for the target CTLA-4 gene and the reference GAPDH gene.

Relative quantitative PCR analysis was performed on ViiA™ 7 real-time PCR system (Thermo Fisher Scientific, Waltham, MA, USA) using the SYBR Green PCR Master Mix (cat no 4385612; Thermo Fisher Scientific, Waltham, MA, USA) set for 40 cycles at 95 °C for 15 s, 60 °C for 1 min/cycle. Primers for CTLA-4 analysis were 5′-ACGGGACTCTACATCTGCAAGG-3′ and 5′-CCCCGAACTAACTGCTGCAA-3′, and for GAPDH were 5′-ACCCACTCCTCCACCTTTGAC and 5′-TCCACCACCCTGTTGCTGTAG-3′ as housekeeping gene. qRT-PCR was performed following the manufacturer’s protocol. Briefly, for each sample, a 3 μL cDNA sample was used with 10 μL of SYBR green Mix and 2 μL primer mix (μL F+ μL R) to a total of 20 μL reaction volume. A negative control free of cDNA was used for each reaction to detect non-specific amplification. All reactions were performed with the same concentration of cDNA per reaction. The product specificity of the primers was evaluated for each reaction through the melting curve. Figure 1 shows the amplification curves and the melting curves of the target CTLA-4 gene and the reference GPADH gene. The melting curves of Figure 1C,D show a single distinct peak for CTLA-4 and GAPDH, respectively. Analysis for relative gene expression was performed using the 2^−ΔΔCT^ method [43]. where ∆Ct = (Ct target gene − Ct GAPDH). The expression of CTLA-4 in was performed on 31 specimens and the gene expression level was normalized relative to GAPDH. Normal tissue samples (30 specimens) were used as a calibrator, and GAPDH as a reference gene for normalization. A two-tailed T test was performed using GraphPad Prism software with significance threshold values of *p* < 0.05. Data are expressed as mean values with SD.

### 2.5. SNP Selection and Genotyping

Three CTLA-4 SNPs were selected: CTLA-4 −658C > T(rs11571317), +49A > G (rs231775) and CT60 G > A (rs3087243) were genotyped using TaqMan allelic discrimination assay following the previously described protocol [44]. TaqMan CTLA-4 SNP Genotyping Assays having catalogue number 4351379 and assay numbers 4351379 C___3296043_10 (rs3087243), 4351379 C__30981396_10 (rs11571317) and 4351379 C___2415786_20 (rs231775) were acquired from Applied Biosystems. These assays were supplied at 40X concentration. For each PCR, a 5 ng DNA sample was used with 10 μL of 2X Universal Master Mix and 1X assay mix in a total 20 μL reaction volume (Applied Biosystems, Foster City, CA, USA). PCR conditions were pre-read stage 60 °C for 30 s, hold stage 95 °C for 10 min, PCR stage 95 °C for 15 s and 60 °C for 1 min for 40 cycles, and post-read stage at 60 °C for 30 s. All genotypes were determined using end-point reading on the ViiATM 7 Real-Time PCR System (Applied Biosystems, Foster City, CA, USA). VIC and FAM were used as a probes for the alleles [VIC/FAM]: rs11571317 [C/T], rs231775 [A/G] and rs3087243 [A/G].

For quality control, 5 % of the samples were selected randomly and repeated analysis were performed for verification procedures. The results were reproducible without any inconsistencies.

### 2.6. Construction of Tissue Microarrays (TMAs) and Immunohistochemistry

Colorectal tissue microarrays (TMA) were constructed and immunohistochemistry was performed as described elsewhere [45]. The hematoxylin and eosin stained sections of FFPE tumor samples were used to identify representative areas of viable tumor tissue and 1 mm diameter needle core biopsies were taken using a manual tissue arrayer (Arraymold Kit D IHCWORLD, Woodstock, MD, USA). Three cores of each tumor were taken in order to ensure that representative tumor parts were examined. Cores were taken from both the invasive border and the central tumor arrays to account for tumor heterogeneity. The cores in the paraffin block were incubated for 30 min at 37 °C to improve adhesion between cores and paraffin of the recipient block. Paraffin TMA blocks were micro-dissected using a Leica semi-automatic microtome and mounted on glass slides.

TMA block sections with 5 m thicknesses were stained using immunohistochemistry. The slides were then washed in Tris-buffered saline (TBS) for 10 min after endogenous peroxidase activity had been quenched with 3% hydrogen peroxide in distilled water for 5 min. Protein Block (Novocastra, Milton Keynes, UK) was used to prevent non-specific antibody binding. Subsequently, the mouse anti-CTLA-4 monoclonal antibody (cat #PA 5-115060) was then applied to the slides (1:100) and incubated for 1 h at room temperature. After washing three times with TBS, biotinylated anti-mouse IgG (Novocastra, Milton Keynes, UK) was incubated for 30 min. Diaminobenzedine (DAB) (Novocastra, Milton Keynes, UK) substrate was used to detect peroxidase. The last step was to counterstain the slides with Mayer’s hematoxylin (Novocastra, Milton Keynes, UK). The negative control was prepared following the same procedure but without primary antibody. The expression of CTLA-4 in tumor and normal tissues was analyzed using the eSlide capture device (ScanScope CS, Aperio Technologies Inc., Vista, CA, USA). The digital slide images were viewed by Aperio’s viewing software version 12.3.3 (ImageScope), and analyzed using Aperio’s image analysis algorithms. High-resolution, whole-slide digital scans of all TMA glass slides were created with a ScanScope slide scanner (Aperio Technologies, Inc. Vista, CA, USA) as previously described [45].

### 2.7. Statistical Analysis

Assessments of genotype and allelic associations between CRC and three CTLA-4 SNPs were assessed using the SNPStats software [46]. Five inheritance models, including co-dominant, dominant, recessive, over-dominant and log-additive models were tested for association, and odds ratios (OR) with 95 % confidence intervals (CI) were calculated using logistic regression. Hardy–Weinberg equilibrium deviancy and χ^2^ values were analyzed through the web-based programs at https://ihg.helmholtz-muenchen.de/cgi-bin/hw/hwa1.pl accessed on 23 November 2022. A *p* value of <0.05 was considered significant. Haplotype analysis and linkage disequilibrium (LD) was conducted using the website https://www.snpstats.net/start.htm accessed on 14 November 2022.

## 3. Results

### 3.1. Demographic Characteristics of Study Population

The baseline characteristics of patients are shown in Table 1. The study included 200 participants, 100 patients with sporadic colorectal cancer and 100 healthy cancer-free individuals without a history of cancer. The patient group consisted of 64 males and 36 females with a mean age of 56.33 ± 14.56 years. The control group consisted of 65 males and 35 females with a mean age of 56.31 ± 14.56 years (age and sex controls). Patients were classified into four TNM stages, I, II, III and IV, where 57% belong to stage I and II (early stage) and 32% to stage III and IV (late stage). For all these subjects, the genotyping of CTLA-4 was performed using TaqMan assay for three selected SNPs; rs11571317, rs231775 and rs3087243 (Table 2). The genetic and allelic association of these polymorphisms with CRC was tested in five genetic models (allelic, codominant, dominant, recessive, over-dominant and additive) as reported in Table 3.

The distribution of the genotype CT60 G > A SNPs in the control group followed HW equilibrium while the −658C > T and +49A > G (*p* ≤ 0.0001) deviated from HWE. The TaqMan genotyping of +49A > G showed that the GG genotype was significantly more frequent in patients (47%) compared to controls (5%) and was associated with high risk to develop CRC in both codominant model (OR = 8.93; 95% CI (3.21–24.84); *p* < 0.0001) and recessive model (OR = 16.32; 95% CI (0.03–0.19); *p* < 0.0001). Conversely, the AG genotype exhibited a protective effect against CRC in both codominant (OR = 0.23; 95% CI (0.11–0.48); *p* < 0.0001), and additive (OR = 1.94; 95% CI (1.34–2.81); *p* = 3 × 10^−4^) models, after applying the Bonferroni correction. The G allele was significantly highly frequent in CRC patients compared to the healthy individual group (0.53 vs. 0.33), suggesting an increased susceptibility to CRC for individuals sharing this allele (OR = 2.337; 95% CI (1.553–3.516); *p* = 0.00004). For the rs3087243 CT60 G > A polymorphism, our analysis showed that the GA genotype was the most frequent in both patients and controls followed by the AA in patients and GG in healthy controls. In the codominant model, the AA genotype was found with higher frequency in CRC (35%) compared to healthy controls (14.1%), and this difference was found to be significant (OR = 3.323; 95% CI (1.408–7.843); *p* = 0.00535). The A allele was associated with higher risk of CRC (OR = 1.816; 95% CI (2.77–1.19); *p* = 0.005). Furthermore, the allele G was the most common allele in comparison with the A allele in patients, which aligns with the global MAF database (A = 0.3690. Our analysis for the rs11571317 −658C > T polymorphism did not show any significant association with CRC for all the studied models (Table 3).

### 3.2. Age and Gender Stratified Analysis

We stratified 100 cases into 2 subgroups to investigate the possible effect of SNPs according to age distribution: CRC ≥ 56 (n = 59) and CRC < 56 years (n = 41). We compared the allelic and genetic distributions of three SNPs between these two groups according to five genetic patterns. Our analysis did not reveal significant associations for all three SNPs (Table 4). A higher frequency of the G allele was observed in patients older than 56 years, but its significance was lost after application of the Bonferroni correction. Furthermore, we divided the 100 cases into 2 subgroups: female (n = 36) and male (n = 64) to investigate the possible effect of SNPs on risk according to gender distribution. We compared the allelic and genetic distributions of the three SNPs between these two groups across the five inheritance patterns (Table 5).

### 3.3. Haplotype Analysis

As shown in Table 6, three SNPs were used to generate haplotypes for the cases and controls. The accumulated frequency of five common haplotypes of rs11571317, rs231775 and rs3087243 exceeded 90%. There were differences between cases and controls in the distribution of haplotypes. As a reference, the A-G-G haplotype was the most common with 20% in cases and 29% in healthy people. In individuals with G-A-G haplotypes, CRC risk was decreased by 57 times (OR = 57.66; 95% CI 6.82–487.84; *p* = 3 × 10^−4^). The global statistical test suggests an association between these haplotypes and CRC (0 < 0.0001).

### 3.4. Gene Expression Analysis

**Figure 2:**
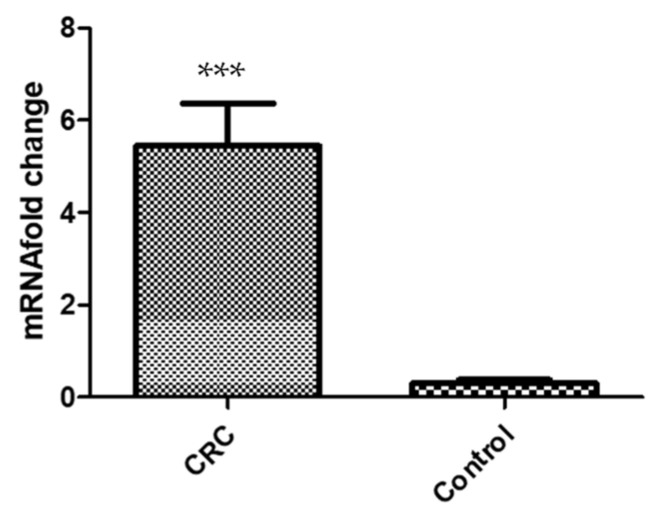
The relative CTLA-4 gene expression was estimated using the 2^−∆∆Ct^ method and normalized to the average of the GAPDH housekeeping gene. (mean ± SE) (*** *p* < 0.001).

Relative quantification of CTLA-4 gene expression was performed by quantitative real-time (q-RT) PCR from 31 colon cancer fresh tissues and 30 normal adjacent matching tissues. The CTLA-4 mRNA was found significantly higher (5–6-fold differences; *p* < 0.001) in tumors (mean = 5.4 ± 0.92; CI = 3.5–7.3) compared to normal adjacent colon samples (mean = 0.30 ± 0.064; CI = 0.17–0.44) (Figure 2).

### 3.5. Protein Expression of CTLA-4 in CRC Patients

**Figure 3:**
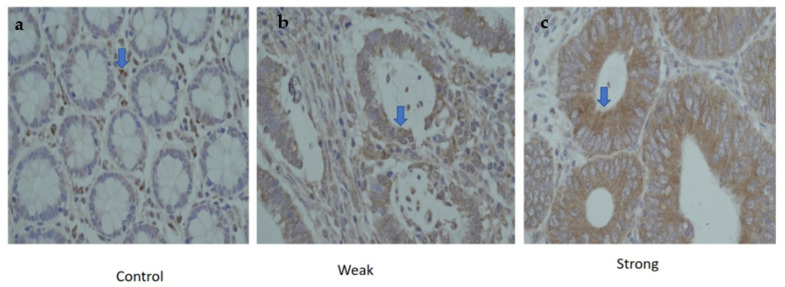
Immunohistochemistry staining for CTLA-4. (Arrows). A group of colonic adenocarcinomas showing strong positive epithelial neoplastic luminal for CTLA-4 in an early-stage patient (**a**) Control. (**b**) Weak expression. (**c**) Strong expression.

CTLA-4 immunohistochemistry was performed on the TMA of paired tumor and adjacent normal specimens (n = 20). Increased expression of CTLA-4 (brown staining) was confirmed in cancer tissues compared to normal tissues (Figure 3a). Early-stage (stages 1 and 2) tumor tissues had higher numbers of CTLA-4-positive tumor cells compared to late stage (stages 3 and 4) (Figure 3a,b). Semiquantitative analysis was also performed with negative, weak or strong staining depending on the intensity of CTLA-4 staining. Weak staining was observed in 47% of late CRC samples, whereas 53% samples showed strong staining in early stage. While all tumors showed CTLA-4 positivity, the intensity of staining varied from weak and/or strong between early (stages I and II) and late stages (stages III and IV) of cancer and within the same stage, demonstrating heterogeneous expression.

## 4. Discussion

In the present study, we have explored the possible correlation between three CTLA-4 SNPs: rs11571317 (−658C > T), rs231775 (+49A > G) and rs3087243 (CT60 G > A), and the risk of CRC in Saudi Arabia. The departure from HWE for two out of three SNPS could be explained by non-random mating because of the highly consanguineous marriages that characterize the Saudi society, causing a deficiency in heterozygous genotypes [47]. Our analyses show strong positive associations of the +49A > G and CT60 polymorphisms with CRC. For the +49A > G polymorphism, strong positive associations were found for the G allele and GG genotype in almost all tested inheritance models (*p* < 0.001). This CTLA-4 genetic variants in the exon 1 is a missense variation leading to a threonine to alanine substitution at codon 17 (Thr17Ala). Functional analysis has shown that this polymorphism result in an inefficient CTLA-4 glycosylation and reduced cell surface expression and consequently disruption of the balance CD28 and CTLA-4 interactions with B7-1/2 [48,49]. In various case reports or systematic review, the +49A > G variant was connected to increased risks to many cancer diseases, including head and neck cancer, breast cancer, lung cancer, esophageal, liver cancer and pancreatic cancer [27,50,51,52]. This variant was associated with an increased risk of CRC [53]. Wang found a significant association between the CTLA-4 A49G polymorphism and CRC risk among an Asian population, but not among Caucasians [54]. Furthermore, a meta-analysis showed a significant association between the +49A/G polymorphism and CRC risk; after subgroup analysis by ethnicity, a significant association in Asian ethnicity was reported but this was not present among Europeans [55]. Conversely, a study performed in Iran did not find statistically significant differences in the genotype distribution and allele frequencies among CRC patients and controls [56]. In addition, in a Turkish population showed no significant association between rs231775 and the risk of gastric cancer [57]. Two meta-analyses found similar results regarding the rs231775 variant, showing an associated risk for cancer development even through subgroup analysis by type and ethnicity suggesting that CTLA-4 rs231775 is a key variant that could be associated with cancer development [58,59]. A previous study on the rs231775 variant involving the Taiwanese population showed that patients with the AA genotype had early onset of German oral squamous cell carcinoma (OSCC) in comparison to AG and GG carriers, and that the G allele might provide an active immune response [38]. Our findings are supported by a study that reported that the absence of the G allele in rs231775 decreases immune response and contributes to peripheral tolerance [60,61]. Individuals with the GG genotype showed 30% less CTLA-4 on their T cell surfaces when compared to the AA genotype [62]. Moreover, in another study, individuals with the AA genotype had lower T cell activity compared to the GG genotype [27]. Furthermore, the rs231775 GG genotype may be associated with a lower risk of breast cancer in Iranian women [25]. The proportion of the AG genotype was higher in patients than in controls, which agrees with a previous meta-analysis that showed that genotypic variation at this locus significantly increased the risk of non-epithelial and epithelial tumors in Caucasian, Asian and Chinese people [63]. In addition, the CTLA-4 49A/G SNP was related to infection-related hepatocellular and cervical carcinomas [51]. Our findings are further supported by Li et al.; they reported that patients carrying the rs231775 AA genotype had a 2.06-fold higher risk for cervical cancer compared to patients with the GG genotype. Several studies reported that the rs231775 G allele increases susceptibility to many autoimmune diseases; however, it is also linked to a lower risk of graft-vs.-host disease after allogeneic transplantation [64], while the A allele has been linked to an increased cancer risk, such as breast cancer and lung cancer [50,58].

On the other hand, the AA and AG genotypes of CT60 were also found positively associated with CRC in codominant, dominant and additive models. For the CTLA-4 gene, few studies have reported an association between the tested SNPs and CRC. The CT60 of the CTLA-4 gene was found associated with the risk of CRC in the Swedish population and patients carrying the A allele were at a higher risk for CRC and the dissemination of the disease [65]. A previous study, however, suggested an association between the G allele of CT60 and risk of breast cancer [28]. Although another study performed on the Han population from northeastern China did not find any association with breast cancer risk [26,66], another pooled analysis on CT60 showed a lower risk for breast cancer [67,68]. A previous meta-analysis reported that the CTLA-4 CT60 is associated with an increased risk of skin cancer [69], while another study showed an increased risk of hepatocellular carcinoma (HCC) [68]. Moreover, reports on European and Asian populations showed an increased risk of rheumatoid arthritis (RA) in individuals carrying the G allele compared to those having the A allele of CT60 [70,71]. Inversely, another study on the Mexican population showed a decreased risk of RA [72] and there was no association found in a Polish population [66,73]. The mechanism and function of CTLA-4 expression and regulation is complex, and more studies are required to confirm the mechanisms by which CTLA-4 expression affects T cell activity [58]. Conflicting results obtained from previous studies could be due to differences in genetic background, environmental exposures, various lifestyles and different experimental designs that could influence results [74,75,76]. Moreover, these environmental factors could cause alterations or epigenetic effects that could alter the DNA structure, causing changes in the level of expression or stability [77,78]. Although it is debatable whether genotype combinations increase cancer risk, in our study, results showed that the GG A haplotype was found only in patients and was associated with CRC risk (OR 57.66; CI (6.82–487.84); *p* value 3 × 10^−4^). Haplotype analysis revealed that the TACG haplotype for the CTLA-4 variants (−1722T, −1661A, −318C, +49G) was significantly associated with an increased risk of CRC and gastric cancer. However, the TACA haplotype in the same study was significantly lower in CRC patients but not in gastric cancer patients [56]. A study found a significantly higher risk among the carriers of CTLA-4 1661AG and 49AA genotypes [79]. Li et al. showed that CTLA-4 haplotype CAAA of the variants 1722C, 1661A, 49A and CT60A had a significant association with progesterone receptor status and high risk of breast cancer [26]. On the other hand, a study by Rahimifar showed that the TGTA haplotype of the CTL4 variants (1722 T, 1661 G, 318 T, 49A) was protective against cervical cancer and that the TGCG haplotype was associated with higher risk [33]. Even after multivariate logistic regression analysis, another study showed an increased risk of OSCC in individuals with the haplotypes TACAG, TGCAA, TATAG and TACGA, while individuals with CACGG and TACGG had a significantly decreased risk [79]. Previous reports have also suggested that the CTLA-4 genotype is an influencing factor, alone or in combination with other T cell regulatory polymorphisms [37,79].

Additionally, we have investigated gene expression in tumor tissue and surrounding normal colon tissue. Our results revealed high expression of the CTLA-4 gene in colorectal cancer tissue as compared to normal tissue. These findings are supported by other reports on the association of CTLA-4 up-regulation and clinical outcomes in several types of cancer including chronic lymphocytic leukemia, breast cancer and CRC [35,80,81]. The CTLA-4 rs231775 SNP causes the substitution of 17Threonine (Thr) to 17Alanine (Ala) (17Thr > 17Ala) in the leading peptide of the CTLA-4 receptor [82], leading to a lower expression of flCTLA-4 on the cell surface. Studies have shown that rs231775 G alleles have lower mRNA efficiency and lower CTLA-4 when compared to the rs231775 A allele [83]. Consequently, carriers of the GG genotype have greater T cell production compared carriers of the AA genotype under suboptimal stimulation [61]. In addition, substitution of 17 Thr to 17Ala in the CTLA-4 rs231775 SNP improves the CTLA-4/B7 interaction. This CTLA-4 17Ala showed more ability to decrease T cell activity compared to the CTLA-4 17Thr [27], suggesting that the 17Thr to 17Ala substitution in CTLA-4 produces a stronger CTLA-4 function in negatively regulating T cell activity [84]. In addition, the A allele of the CTLA-4 rs231775 gene has a higher mRNA efficiency and higher CTLA-4 production compared to the G allele [85]. Accordingly, immunotherapy based on the CTLA-4 blockade has determined its value against several malignancies [86,87].

## 5. Conclusions

In conclusion, this is the first study to evaluate the role of CTLA-4 polymorphisms in the risk of developing colorectal cancer in the Saudi population. This study needs to be evaluated in a larger Saudi population for prediction confirmation and exploring the beneficial role for CTLA-4 as a screening biomarker or immunotherapeutic agent against colorectal cancer. Additional studies are crucial to explore the role of CTLA-4 polymorphisms and their involvement in CTLA-4 expression for an improved utilization in immunotherapy against cancer.

## Figures and Tables

**Table 1:** Demographic and main clinical data of CRC patients and control used for SNPs genotyping.

Characteristics		Cancer (100)	Control (100)
Gender (number)	Male	64	65
	Female	36	35
Age (average ± SD)		56.33 ± 14.56	56.31 ± 14.56
Localization	Colon	40	-
	Recto-sigmoid	60	-
Stage	I	11	-
	II	57	-
	III	32	-
	IV	0	-

**Table 2:** Characteristics of selected polymorphisms involved in the CTLA-4 gene.

SNP ID/Assay ID	Common Name	Chromosome Position	Nucleotide Change	Region	MAF in Human Populations (1000 Genomes Study)				Present Study
					Global	European	South Asian	Qatari	
rs11571317	−658C > T	Chr2:203867285	C/T	promoter	T = 0.024	T = 0.08	T = 0.022	T = 0.078	T = 0.26
rs231775	+49A > G	Chr2:203867991	A/G	Exon 1	G = 0.42	G = 0.36	G = 0.28	G = 0.208	G = 0.33
rs3087243	CT60 G > A	chr2:203874196	G/A	3-’UTR	A = 0.36	A = 0.45	A = 0.59	A = 0.567	A = 0.44

**Table 3:** Distribution of CTLA-4 SNPs genotypes and allele frequencies in colorectal cancer cases and control population. CRC, colorectal cancer; AIC, Akaike information criteria; OR, odds ratio; 95 % CI, 95 % confidence interval. * *p* < 0.05 was considered significant.

Locus	Model	Genotype	CRC (%) N = 99	Controls (%) N = 96	OR (95% CI)	* *p*-Value	AIC
rs11571317 (−658C > T)	Alleles	C	136	143	Ref		
		T	62	49	0.75 (0.483–1.170)	0.20502	
	Codominant	CC	56 (56.6%)	61 (63.5%)	1.00		
		CT	24 (24.2%)	21 (21.9%)	1.24 (0.63–2.48)		275.2
		TT	19 (19.2%)	14 (14.6%)	1.48 (0.68–3.22)	0.57	
	Dominant	CC	56 (56.6%)	61 (63.5%)	1.00		
		CT + TT	43 (43.4%)	35 (36.5%)	1.34 (0.75–2.38)	0.32	273.3
	Recessive	CC + CT	80 (80.8%)	82 (85.4%)	1.00		
		TT	19 (19.2%)	14 (14.6%)	1.39 (0.65–2.96)	0.39	273.5
	Overdominant	CC + TT	75 (75.8%)	75 (78.1%)	1.00		
		CT	24 (24.2%)	21 (21.9%)	1.14 (0.59–2.23)	0.69	274.1
	Log-Additive				1.22 (0.84–1.770	0.29	273.2
**Locus**	**Model**	**Genotype**	**CRC (%)** **N = 100**	**Controls (%)** **N = 97**	**OR (95% CI)**	* *p*-Value	**AIC**
rs231775 (+49A > G)	Alleles	A	0.46	0.67	Ref		-
		G	0.54	0.33	2.337 (1.553–3.516)	<0.0001	
	Codominant	AA	40 (40%)	38 (39.2%)	1.00	1	
		AG	13 (13%)	54 (55.7%)	0.23 (0.11–048)	<0.0001	212.9
		GG	47 (47%)	5 (5.2%)	8.93 (3.21–24.84)		
	Dominant	AA	40 (40%)	38 (39.2%)	1.00		
		AG + GG	60 (60%)	59 (60.8%)	0.97 (0.55–1.71)	0.91	277
	Recessive	AG + AA	53 (53%)	92 (94.8%)	1.00		
		GG	47 (47%)	5 (5.2%)	16.32 (6.11–43.56)	<0.0001	227.3
	Overdominant	AA-GG	45 (46.4%)	86 (86%)	1.00		235
		AG	13 (13%)	54 (55.7%)	0.12 (0.06–0.24)	<0.0001	
	Log-Additive				1.94 (1.34–2.81)	<0.0001	263.9
**Locus**	**Model**	**Genotype**	**CRC (%)** **N = 97**	**Controls (%)** **N = 99**	**OR (95% CI)**	* *p*-Value	**AIC**
rs3087243 (CT60 G > A)	Alleles	G	0.42	0.56	1		
		A	0.58	0.44	1.743 (1.168–2.599)	0.00631	
	Codominant	GG	19 (19.6%)	26 (26.3%)	1.00		
		GA	44 (45.4%)	59 (59.6%)	1.021 (0.502–2.073)	0.95521	265.8
		AA	34 (35%)	14 (14.1%)	3.323 (1.408–7.843)	0.00535	
	Dominant	GG	19 (19.6%)	26 (26.3%)	1.00		
		GA + AA	78 (80.4%)	73 (73.7%)	1.462 (0.747–2.864)	0.26660	263.8
	Recessive	GG + GA	63 (65%)	85(85.9%)	1.00		
		AA	34 (35%)	14 (14.1%)	0.305 (0.151–0.616)	0.00067	274.5
	Overdominant	AA + GG	53 (54.6%)	40 (40.4%)	1.00		
		AG	44 (45.4%)	59 (59.6%)	0.56 (0.32–0.99)	0.046	271.7
	Log-Additive				1.816 (1.19–2.77)	0.0050	267.7

**Table 4:** Association of CTLA-4 with colorectal cancer cases after age stratification. CRC, colorectal cancer; OR, odds ratio; 95 % CI, 95 % confidence interval. * *p* < 0.05 was considered significant.

Locus	Model	Genotype	CRC < 56 (%) N = 41	CRC > 56 (%) N = 58	OR (95% CI)	* *p*-Value	AIC
rs11571317 C > T	Allele	C	0.67	0.7	Ref		
		T	0.33	0.3	0.880 (0.479–1.616)	0.68059	
	Codominant	CC	23 (56.1%)	33 (56.9%)	1.00		
		CT	9 (21.9%)	15 (25.9%)	1.22 (0.45–3.29)	0.79	140.7
		TT	9 (21.9%)	10 (17.2%)	0.79 (0.28–2.26)		
	Dominant	CC	23 (56.1%)	33 (56.9%)	1.00		139.2
		CT + TT	18 (43.9%)	25 (43.1%)	1 (0.44–2.26)	1	
	Recessive	CC + CT	32 (78 %)	48 (82.8%)	1.00		
		TT	9 (21.9%)	10 (17.2%)	0.74 (0.27–2.05)	0.57	138.8
	Overdominant	CC + TT	32 (78%)	43 (74.1%)	1.00		
		CT	9 (21.9%)	15 (25%)	1.29 (0.50–3.36)	0.59	138.9
	Log-Additive				0.93 (0.56–1.55)	0.78	139.1
**Locus**	**Model**	**Genotype**	**CRC < 56 (%)** **N = 41**	**CRC > 56 (%)** **N = 59**	**OR (95% CI)**	* ***p*-Value**	**AIC**
rs231775 A > G	Allele	A	0.38	0.53	Ref		
		G	0.62	0.47	0.549 (0.309–0.975)	0.03986	
	Codominant	AA	13 (31.7%)	27 (45.8%)	2.02 (0.84–4.86)		
		AG	5 (12.2%)	8 (13.5%)	1.50 (0.43–5.31)	0.28	140
		GG	23 (56.1%)	24 (40.7%)	1.00		
	Dominant	GG	13 (31.7%)	27 (45.8%)	1.00		
		AG-AA	28 (68.3%)	32 (54.2%)	1.87 (0.83–4.21)	0.13	138.2
	Recessive	GG-AG	18 (43.9%)	35 (59.3%)	1.00		
		AA	23 (56.1%)	24 (40.7%)	1.85 (0.80–4.28)	0.15	138.4
	Overdominant	AA-GG	36 (87.8%)	51 (87.5%)	1.00		
		AG	5 (12.2%)	8 (13.5%)	1.10 (0.33–3.67)	0.87	140.4
	Log-Additive				1.42(0.92–2.21)	0.11	138
**Locus**	**Model**	**Genotype**	**CRC < 56 (%)** **N = 40**	**CRC > 56 (%)** **N = 57**	**OR (95% CI)**	* ***p*-Value**	**AIC**
rs3087243 G > A	Allele	G	39	43	Ref		
		A	41	71	1.571 (0.880–2.803)	0.12576	
	Codominant	GG	12 (30%)	7 (12.3%)	0.38 (0.12–1.22)		
		AG	15 (37.5%)	29 (50.9%)	3.314 (1.080–10.172)	0.03238	134.2
		AA	13(32.5%)	21 (36.8%)	2.769 (0.867–8.840)	0.08133	
	Dominant	AA	12 (30%)	7 (12.3%)	1.00		
		AG-GG	28 (70%)	50 (87.7%)	3.061 (1.081–8.666)	0.03042	136.1
	Recessive	AA-AG	27 (67.5%)	36 (63.2%)	1.00		
		GG	13(32.5%)	21 (36.8%)	0.825 (0.352–1.937)	0.65906	132.3
	Overdominant	GG-AA	25 (62.5%)	28 (49.1%)	1.00		
		AG	15 (37.5%)	29 (50.9%)	1.65 (0.72–3.79)	0.24	134.9
	Log-Additive				0.67 (0.38–1.19)	0.17	134.9

**Table 5:** Association of CTLA-4 with colorectal cancer cases after gender stratification. CRC, colorectal cancer; OR, odds ratio; 95 % CI, 95 % confidence interval. * *p* < 0.05 was considered significant.

Locus	Model	Genotype	CRC Female (%) N = 34	CRC Male (%) N = 62	OR (95% CI)	* *p*-Value	AIC
rs11571317 C > T	Alleles	C	0.66	0.7	Ref		
		T	0.34	0.3	0.81 (0.43–1.51)	0.505	
	Codominant	CC	18 (51.4%)	38 (59.4%)	1.00		
		CT	10 (28.6%)	14 (21.9%)	0.66 (0.25–1.78)	0.71	133.9
		TT	7 (20%)	12 (18.8%)	0.81 (0.27–2.41)		
	Dominant	CC	18 (51.4%)	38 (59.4%)	1.00		
		CT + TT	17 (48.6%)	26 (40.6%)	0.72 (0.32–1.66)	0.45	132
	Recessive	CC + CT	28 (80%)	52 (81.2%)	1.00		
		TT	7 (20%)	12 (18.8%)	0.92 (0.32–2.61)	0.88	132.6
	Overdominant	CC + TT	25 (71.4%)	50 (78.1%)	1.00		
		CT	10 (28.6%)	14 (21.9%)	0.70 (0.27–1.80)	0.46	132.1
	Log-Additive				0.86 (0.60–1.25)	0.58	132.3
**Locus**	**Model**	**Genotype**	**CRC Female (%)** **N = 36**	**CRC Male (%)** **N = 64**	**OR (95% CI)**	* *p*-Value	**AIC**
rs231775 A > G	Alleles	A	0.47	0.46	Ref		-
		G	0.53	0.54	1.05 (0.59–1.87)	0.88	
	Codominant	AA	15 (41.7%)	25 (39.1%)	0.94 (0.39–2.26)		
		AG	4 (11.1%)	9 (14.1%)	1.27 (0.34–4.77)	0.91	136.5
		GG	17 (47.2%)	30 (46.9%)	1		
	Dominant	GG	17 (47.2%)	30 (46.9%)	1.00		
		AG + AA	19 (52.8%)	34 (53.1%)	1.01 (0.45–2.30)	0.97	134.7
	Recessive	AG + GG	21 (58.3%)	39 (60.9%)	1.00		
		AA	15 (41.7%)	25 (39.1%)	0.90 (0.39–2.06)	0.8	134.6
	Overdominant	AA-GG	32 (88.9%)	55 (85.9%)	1.00	0.67	
		AG	4 (11.1%)	9 (14.1%)	1.31 (0.37–4.60)		134.5
	Log-Additive				0.97 (0.63–1.51)	0.91	134.7
**Locus**	**Model**	**Genotype**	**CRC Female (%)** **N = 34**	**CRC Male (%)** **N = 64**	**OR (95% CI)**	* *p*-Value	**AIC**
rs3087243 G > A	Alleles	A	0.53	0.6	Ref		
		G	0.47	0.4	1.365 (0.756–2.466)	0.30173	
	Codominant	AA	10 (28.6%)	9 (14.5%)	1		
		AG	13 (37.1%)	31 (50%)	2.650 (0.874–8.034)	0.08068	129.8
		GG	12 (34.3%)	22 (35.5%)	2.037 (0.650–6.386)	0.21929	
	Dominant	AA	10 (28.6%)	9 (14.5%)	1		
		AG + GG	25 (71.4%)	53 (85.5%)	2.356 (0.851–6.522)	0.09392	130.8
	Recessive	AG + AA	23 (65.7%)	40 (64.5%)	1		
		GG	12 (34.3%)	22 (35.5%)	0.949 (0.397–2.265)	0.90545	128.1
	Overdominant	AA-GG	22 (62.9%)	31 (50%)	1		
		AG	13 (37.1%)	31 (50%)	1.69 (0.73–3.95)	0.22	129.4
	Log-Additive				0.75 (0.42–1.33)	0.32	129.9

**Table 6:** Haplotype analysis. CRC, colorectal cancer; OR, odds ratio; 95 % CI, 95 % confidence interval. * *p* < 0.05 was considered significant.

Rs11571317	Rs231775	Rs3087243	CRC	Control	OR (95% CI)	* *p*-Value
A	G	G	0.1971	0.2973	Ref	
A	A	G	0.2066	0.2357	0.57 (0.26–1.25)	0.16
G	G	G	0.1518	0.2043	1.16 (0.58–2.33)	0.68
A	G	A	0.116	0.1332	1.44 (0.55–3.79)	0.46
G	A	G	0.1364	0	57.66 (6.82–487.84)	3 × 10^−4^

## Data Availability

All data relevant to the study are included in the article.
